# High neutrophil-to-lymphocyte ratio predicts short survival duration in amyotrophic lateral sclerosis

**DOI:** 10.1038/s41598-019-57366-y

**Published:** 2020-01-16

**Authors:** Seok-Jin Choi, Yoon-Ho Hong, Sung-Min Kim, Je-Young Shin, Young Ju Suh, Jung-Joon Sung

**Affiliations:** 1 Department of Neurology, Inha University School of Medicine, Incheon, Republic of Korea; 2 Department of Neurology, Seoul Metropolitan Government Seoul National University Boramae Medical Center, Seoul, Republic of Korea; 3 Department of Neurology, Seoul National University College of Medicine, Seoul, Republic of Korea; 4 Department of Biomedical Sciences, Inha University School of Medicine, Incheon, Republic of Korea

**Keywords:** Prognostic markers, Amyotrophic lateral sclerosis

## Abstract

The present study aimed to investigate the prognostic importance of the neutrophil-to-lymphocyte ratio (NLR) in patients with amyotrophic lateral sclerosis (ALS). Among 322 patients diagnosed as having definite, probable, or possible ALS at a single tertiary hospital, 194 patients were included in the final analysis. Patients were divided into three groups (T1, T2, and T3) according to the tertile of their NLR. Survival rate was significantly lower in T3 compared to the other groups (log-rank test; T1 vs. T3, p = 0.009; T2 vs. T3, p = 0.008). Median survival duration was 37.0 (24.0–56.0), 32.5 (19.5–51.2), and 22.0 (17.0–38.0) months in T1, T2, and T3, respectively. In a multivariable Cox proportional hazards regression analysis, the hazard ratio of age at onset, bulbar-onset, and NLR (T3/T1) was 1.04 (1.02–1.06, p < 0.001), 1.68 (1.10–2.57, p = 0.015), and 1.60 (1.01–2.51, p = 0.041), respectively. A high baseline NLR may serve as a useful indicator for short survival duration in patients with ALS.

## Introduction

Amyotrophic lateral sclerosis (ALS) is a neurodegenerative disease mainly but not exclusively affecting motor neurons in the cerebral cortex, brainstem, and spinal cord. Although the clinical presentation of the disease varies widely between patients, median survival is 3–5 years^1^. The pathophysiology of ALS includes oxidative stress, glutamate excitotoxicity, altered protein homeostasis, defects in RNA processing, impaired axonal transport, and mitochondrial dysfunction^2^, which ultimately impacts the immune system. Accumulating evidence suggests that a dysregulated immune response is an important contributor to the clinical heterogeneity of ALS^3^.

Motor neuron degeneration in ALS occurs both cell-autonomously within motor neurons and non-cell-autonomously involving non-neuronal cells such as astrocytes and microglia^4,5^. Interestingly, alterations in peripheral immune cells as well as glial cells in the central nervous system (CNS) have been reported in humans and mouse models of ALS^6–11^. Leukocyte alterations in easily accessible peripheral blood may therefore be useful disease biomarkers. A recent study showed that an increased neutrophil to CD16- monocyte ratio was associated with disease progression in patients with ALS^12^. In contrast, another study reported that changes in peripheral immune cells, particularly increased neutrophils and decreased CD4 + T-cells, had a significant correlation with disease progression, whereas monocyte count did not^7^.

The neutrophil-to-lymphocyte ratio (NLR) can be calculated by dividing the absolute number of neutrophils by the absolute number of lymphocytes from a simple blood test. The NLR has been shown to be effective in predicting the prognosis of cancer^13^, cardiovascular disease^14^, and cerebrovascular disease^15^ as well as infection^16^. In addition, a significant increase in NLR was reported in patients with Alzheimer’s disease^17^, although its clinical importance has not yet been elucidated^18^. To the best of our knowledge, the prognostic value of the NLR has not been systematically investigated in ALS. In the present study, we hypothesised that the NLR reflects the degree of neuroinflammation in patients with ALS and can be used as a prognostic biomarker for survival.

## Materials and Methods

### Study patients

**Figure 1:**
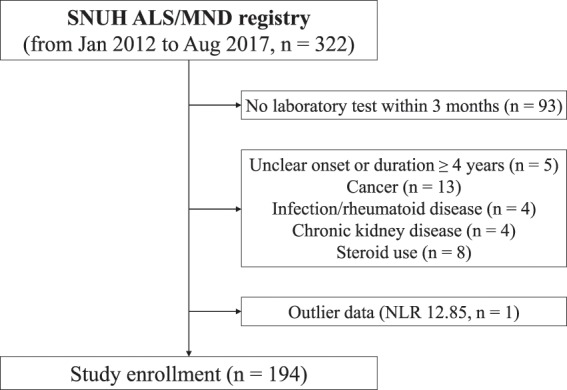
Flow diagram for patient selection.

A total of 322 patients with ALS were identified at the ALS clinic of Seoul National University Hospital between January 2012 and August 2017. All patients fulfilled the criteria for definite, probable, or possible ALS according to the revised El Escorial criteria^19^. The ALS Functional Rating Scale-Revised (ALSFRS-R) score was used to assess the patients’ functional status. Patients were excluded if the blood test was not performed within 3 months before or after the initial clinical assessment (n = 93); onset of disease was unclear or disease duration was longer than 4 years (n = 5); they had any condition associated with NLR changes, such as cancer (n = 13), infectious or rheumatoid disease (n = 4), or chronic kidney disease (n = 4); or they were taking steroids (n = 8). After removing outlier data (one patient with an NLR of 12.85), 194 patients were included in the final analysis (Fig. 1).

The present study was approved by the ethics committee of Seoul National University Hospital (IRB No. 1710-006-890). Requirement for informed consent was waived by the IRB because of the retrospective nature of the study.

### Clinical and laboratory data

Detailed clinical data were retrospectively collected from the medical records and included sex, age of onset, height, weight, comorbidities, onset region, disease duration, forced vital capacity (FVC), ALSFRS-R score, riluzole use, and the date of tracheostomy-invasive ventilation (TIV). The initial progression rate was calculated as follows: (48 – ALSFRS-R score at initial assessment)/(disease duration from symptom onset to initial assessment [months]). The endpoint was defined by either TIV or death. Collected laboratory data included white blood cell (neutrophil, lymphocyte, and monocyte) count, platelet count, and serum levels of C-reactive protein (CRP), uric acid, total protein, albumin, total cholesterol, and creatinine. Information regarding the date of death was obtained from the Ministry of Government Administration and Home Affairs, Republic of Korea.

### Statistical analysis

Patients were divided into three groups according to the tertile of NLR. A comparative analysis between groups was performed using one-way ANOVA with Tukey’s HSD tests for continuous variables and Pearson’s chi-square tests with Bonferroni correction for categorical variables. Wilcoxon signed rank test was used to compare the NLR change according to disease progression. Spearman correlation analysis was used to assess the relationship between the NLR and CRP levels. Kaplan-Meier survival analysis and the log-rank test were used to analyse the prognostic value of the NLR for survival. Univariable and multivariable survival analyses using the Cox proportional hazards (PH) regression model were conducted; variables included were age of onset (years), onset region (bulbar), body mass index (kg/m^2^), forced vital capacity (<80%), and serum level of uric acid (mg/dL). Variables with a p-value (p) less than 0.10 in the univariable analysis were chosen for covariates in the multivariable analysis. A two-tailed p < 0.05 was considered statistically significant. All statistical analyses were performed using the R software (version 3.6.0).

## Results

Table 1 summarises clinical characteristics and results of laboratory tests in each tertile group of NLR. The male to female ratio was not significantly different between groups. The age of onset was younger in the 2nd tertile compared to the other groups, but statistical significance was only met in the comparison between the 2nd and 3rd tertile (T2 vs. T3, 58.0 ± 9.64 vs. 63.9 ± 11.6 years, p = 0.009). The proportion of bulbar-onset ALS, duration of disease, and disease severity measured by ALSFRS-R scores were not significantly different between groups. The initial rate of disease progression seems to be faster in the 3rd tertile compared to the 1st tertile, but there was no statistical significance. The NLR was higher in fast progressors (ALSFRS-R change $\ge$ 1 point/month) compared to slow progressors, but the difference was not statistically significant (fast vs. slow, 2.03 ± 0.82 vs. 1.86 ± 0.97, p = 0.052). There were no significant differences in BMI between groups. Respiratory function as measured by FVC% of predicted was significantly worse in the 3rd tertile compared to the 2nd tertile (FVC < 80%; T2 vs. T3, 26.5% vs. 52.3%, p = 0.024). The mean FVC% was 74.1 ± 19.3, 78.8 ± 20.0, and 72.4 ± 22.4 in the 1st, 2nd, and 3rd tertiles, respectively. There were no significant differences observed in serum levels of CRP, uric acid, total cholesterol, albumin, and creatinine between the groups. There was no significant correlation between the NLR and CRP levels (rho = 0.098, p = 0.193).

**Table 1:** Comparison of clinical characteristics and results of laboratory tests between NLR tertile groups.

Variables	T1 (n = 65)	T2 (n = 64)	T3 (n = 65)	P-value^*^			
				Overall	T1 vs. T2	T1 vs. T3	T2 vs. T3
NLR^a^	1.14 ± 0.26	1.77 ± 0.19	2.91 ± 0.85	<0.001	<0.001	<0.001	<0.001
Male/Female^b^	32/33	38/26	39/26	0.381	NS	NS	NS
Age at onset (years)^a^	61.5 ± 12.2	58.0 ± 9.64	63.9 ± 11.6	0.012	NS	NS	0.009
BMI (kg/m^2^)^a^	22.8 ± 2.51	22.9 ± 2.80	22.1 ± 3.28	0.282	NS	NS	NS
Onset region^b^				0.071	NS	NS	NS
Bulbar	23	11	17				
Limb	42	52	47				
Others	0	1	1				
From onset to diagnosis (months)^a^	13.7 ± 7.93	14.0 ± 9.22	13.4 ± 8.81	0.906	NS	NS	NS
ALSFRS-R^a^	39.7 ± 6.55	38.6 ± 5.67	38.5 ± 6.18	0.562	NS	NS	NS
Progression rate^c^	0.56 (0.30–0.80)	0.61 (0.33–1.21)	0.66 (0.40–1.27)	0.142	NS	NS	NS
FVC^b^				0.017	NS	NS	0.024
≥80%	20	27	16				
<80%	23	17	34				
Not performed	22	20	15				
Riluzole use^b^	9	17	16	0.166	NS	NS	NS
Total cholesterol (mg/dL)^a^	188 ± 38.1	191 ± 36.7	185 ± 32.0	0.631	NS	NS	NS
Albumin (g/dL)^a^	4.15 ± 0.26	4.15 ± 0.26	4.09 ± 0.32	0.388	NS	NS	NS
Creatinine (mg/dL)^a^	0.67 ± 0.15	0.69 ± 0.14	0.69 ± 0.16	0.678	NS	NS	NS
Uric acid (mg/dL)^a^	4.89 ± 1.47	5.08 ± 1.50	4.70 ± 1.37	0.330	NS	NS	NS
CRP (mg/dL)^c^	0.07 (0.02–0.14)	0.06 (0.02–0.12)	0.07 (0.02–0.17)	0.242	NS	NS	NS
Survival duration (months)^c^	37.0 (24.0–56.0)	32.5 (19.5–51.2)	22.0 (17.0–38.0)	NA	NA	NA	NA

^a^Data are expressed as mean ± standard deviation. ^b^Data are expressed as the number of patients. ^c^Data are expressed as median (1Q–3Q).

^*^One-way ANOVA with Tukey’s HSD tests for continuous variables or Pearson’s chi-square tests with Bonferroni correction for categorical variables.

Abbreviations: NLR = neutrophil-to-lymphocyte ratio; BMI = body mass index; ALSFRS-R = ALS functional rating scale-revised; FVC = forced vital capacity; CRP = C-reactive protein; NS = not significant (p-value ≥ 0.1); NA = not applicable.

**Figure 2:**
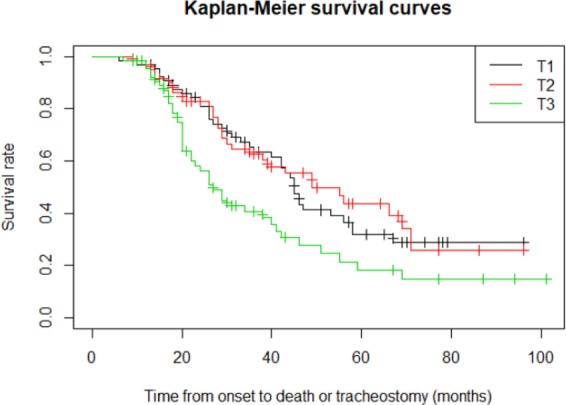
Kaplan-Meier survival curves. The survival rate was significantly lower in the 3rd tertile compared to the other groups (log-rank test; T1 vs. T3, p = 0.009; T2 vs. T3, p = 0.008).

The survival rate estimated by the Kaplan-Meier survival analysis was significantly lower in the 3rd tertile compared to the other groups (log-rank test; T1 vs. T3, p = 0.009; T2 vs. T3, p = 0.008; Fig. 2). The median survival duration was 37.0 (24.0–56.0), 32.5 (19.5–51.2), and 22.0 (17.0–38.0) months in the 1st, 2nd, and 3rd tertiles, respectively. Table 2 shows the results of univariable and multivariable Cox PH regression analyses for survival. In a multivariable Cox PH regression analysis, the hazard ratio (95% confidence interval) of age at onset, bulbar-onset, and 3rd/1st tertile of NLR was 1.04 (1.02–1.06, p < 0.001), 1.68 (1.10–2.57, p = 0.015), and 1.60 (1.01–2.51, p = 0.041), respectively.

**Table 2:** Univariable and multivariable Cox proportional-hazards regression analyses for survival.

Variables	Univariable		Multivariable	
	HR (95% CI)	P-value	HR (95% CI)	P-value
Age (years)	1.05 (1.03–1.07)	<0.001	1.04 (1.02–1.06)	<0.001
Onset region (bulbar)	1.92 (1.27–2.90)	0.001	1.68 (1.10–2.57)	0.015
BMI (kg/m^2^)	0.96 (0.89–1.02)	0.239		
FVC (<80%)	1.40 (0.88–2.22)	0.145		
Uric acid (mg/dL)	0.94 (0.82–1.07)	0.374		
NLR (T3/T1)	1.79 (1.15–2.79)	0.009	1.60 (1.01–2.51)	0.041
NLR (T2/T1)	0.90 (0.56–1.46)	0.693	1.08 (0.66–1.75)	0.749

Abbreviations: HR = hazard ratio; CI = confidence interval; BMI = body mass index; FVC = forced vital capacity; NLR = neutrophil-to-lymphocyte ratio.

The NLR had increased at subsequent visits as the disease progresses: follow-up NLR was significantly greater than baseline NLR (baseline vs. follow-up NLR, 2.06 ± 1.13 vs. 3.04 ± 1.81, p < 0.001) and the average rate of NLR change per month was + 0.175 ± 0.804.

We further analysed the prognostic value of the neutrophil-to-monocyte ratio (NMR). The NMR was also higher in fast progressors compared to slow progressors, but the difference was not statistically significant (fast vs. slow, 8.96 ± 3.16 vs. 8.05 ± 2.86, p = 0.079). The NMR was positively correlated with NLR (rho = 0.498, p < 0.001), but was not associated with CRP levels (rho = 0.019, p = 0.793). In survival analysis using tertile stratification, high NMR did not predict short survival length (data not shown).

## Discussion

The present study examined the prognostic significance of the baseline NLR in patients with ALS. We showed that a high NLR indicates short survival duration in ALS. While a recent study suggested elevated serum CRP as a prognostic biomarker for predicting rapid progression and short survival^20^, the NLR was not correlated with serum CRP levels in our analysis.

A close relationship between the NLR and disease progression of ALS is supported by several previous studies. First, an increase in peripheral blood neutrophils has been repeatedly reported in patients with ALS^21–24^. More recently, a significant correlation between the neutrophil increase in peripheral blood and disease progression of ALS was demonstrated^7,12^. Neutrophils have a proinflammatory role in neurodegeneration and contribute to the breakdown of the blood-spinal cord barrier^25,26^. Alternatively, neutrophils may have a protective role in initiating the neuronal repair; thus, an increase in blood neutrophils may reflect the exclusion of these cells following initiation of the repair process^27–29^. Since short-lived neutrophils are produced in the bone marrow (BM) and released into the bloodstream, an increased neutrophil population in peripheral blood is possibly caused by increased BM production rather than reduced CNS recruitment. Second, perivascular and intraparenchymal T-lymphocytes were found in proximity to degenerating corticospinal tracts and ventral horns in two-thirds of ALS autopsy cases^30^. In mouse studies of ALS, CD4 + T-lymphocytes and M2 microglia/macrophages actively contribute to neuroprotection in the early neuroprotective phase of the disease, whereas in the late cytotoxic phase, the deleterious response of CD8 + T-lymphocytes and M1 microglia/macrophages and suppression of regulatory T-lymphocytes are major contributors to the motor neuron degeneration^3,31^. Flow cytometric analyses showed that reduced CD4 + T-lymphocytes in peripheral blood were associated with rapid progression in patients with ALS^7^. Survival duration of ALS patients was positively correlated with peripheral lymphocyte count in a univariate analysis; however, there was no significant correlation between them after adjusting for the covariates^23^. Although peripheral blood lymphocytes are composed of various B- and T-lymphocyte subsets, the most abundant subtype is CD4 + T-lymphocytes^32^. The reduced lymphocyte population in peripheral blood may therefore indicate the recruitment of T-lymphocytes to the CNS.

Silent infection, such as pneumonia resulting from microaspiration, which may increase the NLR, might contribute to the results of this study as confounding factors. Nonetheless, serum levels of CRP, a strong indicator for systemic inflammation or infection, were not markedly elevated in all three groups and were not significantly different between the groups. Additionally, there was no significant correlation between the NLR and CRP levels in a correlation analysis. Although a recent cohort study showed that elevated serum CRP levels at initial assessment were associated with rapid progression and short survival in patients with ALS^20^, neutrophil and lymphocyte counts in peripheral blood were not analysed. Therefore, our study suggests that increased NLR may not be a consequence of systemic inflammation, but is rather due to the specific immune modulations that underlie the pathogenesis of ALS. A high NLR may indicate increased neutrophil production in the BM and/or T-lymphocyte recruitment to the CNS, thereby contributing to the clinical heterogeneity of ALS. Interestingly, in contrast to a previous study in which NLR did not seem to increase over time^24^, our results showed that the NLR significantly increased at the subsequent visits with disease progression. It may be caused by the inflammation following motor neuron degeneration as well as aforementioned immune modulations.

Of note, the results of the present study should be interpreted with caution. The specific cut-off value of the NLR for rapid disease progression might not be generalised into other ALS studies with different race, sex, and age groups. Although a recent study involving South Korean healthy adults showed that the mean NLR across all age groups was 1.65^33^, there are no standardised reference values for the NLR. The NLR in the Asian population was reported to be generally lower than that in Western countries^33^, and the cut-off values of the NLR for predicting the prognosis of diseases widely vary between the studies^13–15,17^. Accordingly, we suggest that the prognostic role of a high NLR in patients with ALS should be considered in the context of its distribution within each cohort.

There are several potential limitations to the present study. First, as this was a retrospective study conducted at a single national central hospital, the number of patients was relatively small and blood samples could not be obtained at the earliest stages of the disease. Second, the current analysis for survival did not include several known prognostic factors such as C9orf72 repeat expansion and progression rate^34^. Although genetic studies were not performed in our patients, unlike in Caucasian populations, C9orf72 repeat expansion is reported to be very rare in Korean ALS patients^35^. Initial progression rate was not significantly different between groups, however, subgroup analysis (fast vs. slow progressors) may be needed to more robustly investigate the prognostic significance of NLR. Lastly, alterations in peripheral immune cells may be a consequence of motor neuron degeneration rather than a contributor to disease progression.

In conclusion, the results of this study suggest that an elevated baseline NLR is a useful and easily accessible indicator for short survival duration in patients with ALS. Further studies involving a larger number of patients and serial measurements of the NLR are warranted.

## Data Availability

The datasets generated during and/or analysed during the current study are available from the corresponding author on reasonable request.

## References

[1] Swinnen B, Robberecht W (2014). The phenotypic variability of amyotrophic lateral sclerosis. Nat. Rev. Neurol..

[2] Brown RH, Al-Chalabi A (2017). Amyotrophic Lateral Sclerosis. N. Engl. J. Med..

[3] Beers DR, Appel SH (2019). Immune dysregulation in amyotrophic lateral sclerosis: mechanisms and emerging therapies. Lancet Neurol..

[4] Ilieva H, Polymenidou M, Cleveland DW (2009). Non-cell autonomous toxicity in neurodegenerative disorders: ALS and beyond. J. Cell Biol..

[5] Philips T, Robberecht W (2011). Neuroinflammation in amyotrophic lateral sclerosis: role of glial activation in motor neuron disease. Lancet Neurol..

[6] Gustafson MP (2017). Comprehensive immune profiling reveals substantial immune system alterations in a subset of patients with amyotrophic lateral sclerosis. PLoS One.

[7] Murdock BJ (2017). Correlation of Peripheral Immunity With Rapid Amyotrophic Lateral Sclerosis Progression. JAMA Neurol..

[8] Chiu IM (2008). T lymphocytes potentiate endogenous neuroprotective inflammation in a mouse model of ALS. Proc. Natl Acad. Sci. USA.

[9] Henkel JS (2013). Regulatory T-lymphocytes mediate amyotrophic lateral sclerosis progression and survival. EMBO Mol. Med..

[10] Zondler L (2016). Peripheral monocytes are functionally altered and invade the CNS in ALS patients. Acta Neuropathol..

[11] Zhao W (2017). Characterization of Gene Expression Phenotype in Amyotrophic Lateral Sclerosis Monocytes. JAMA Neurol..

[12] Murdock BJ (2016). Increased ratio of circulating neutrophils to monocytes in amyotrophic lateral sclerosis. Neurol. Neuroimmunol. Neuroinflamm.

[13] Templeton AJ (2014). Prognostic role of neutrophil-to-lymphocyte ratio in solid tumors: a systematic review and meta-analysis. J. Natl Cancer Inst..

[14] Dentali F (2018). Impact of neutrophils to lymphocytes ratio on major clinical outcomes in patients with acute coronary syndromes: A systematic review and meta-analysis of the literature. Int. J. Cardiol..

[15] Tokgoz S, Keskin S, Kayrak M, Seyithanoglu A, Ogmegul A (2014). Is neutrophil/lymphocyte ratio predict to short-term mortality in acute cerebral infarct independently from infarct volume?. J. Stroke Cerebrovasc. Dis..

[16] Russell CD (2019). The utility of peripheral blood leucocyte ratios as biomarkers in infectious diseases: A systematic review and meta-analysis. J. Infect..

[17] Kuyumcu ME (2012). The evaluation of neutrophil-lymphocyte ratio in Alzheimer’s disease. Dement. Geriatr. Cogn. Disord..

[18] Rembach A (2014). An increased neutrophil-lymphocyte ratio in Alzheimer’s disease is a function of age and is weakly correlated with neocortical amyloid accumulation. J. Neuroimmunol..

[19] Brooks BR, Miller RG, Swash M, Munsat TL (2000). El Escorial revisited: revised criteria for the diagnosis of amyotrophic lateral sclerosis. Amyotroph. Lateral Scler. Other Mot. Neuron Disord..

[20] Lunetta C (2017). Serum C-Reactive Protein as a Prognostic Biomarker in Amyotrophic Lateral Sclerosis. JAMA Neurol..

[21] Desport JC (2001). Factors correlated with hypermetabolism in patients with amyotrophic lateral sclerosis. Am. J. Clin. Nutr..

[22] Banerjee R (2008). Adaptive immune neuroprotection in G93A-SOD1 amyotrophic lateral sclerosis mice. PLoS One.

[23] Chio A (2014). Amyotrophic lateral sclerosis outcome measures and the role of albumin and creatinine: a population-based study. JAMA Neurol..

[24] Keizman D (2009). Low-grade systemic inflammation in patients with amyotrophic lateral sclerosis. Acta neurologica Scandinavica.

[25] Weiss SJ (1989). Tissue destruction by neutrophils. N. Engl. J. Med..

[26] Garbuzova-Davis S, Sanberg PR (2014). Blood-CNS Barrier Impairment in ALS patients versus an animal model. Front. Cell Neurosci..

[27] Butterfield TA, Best TM, Merrick MA (2006). The dual roles of neutrophils and macrophages in inflammation: a critical balance between tissue damage and repair. J. Athl. Train..

[28] Kim CF, Moalem-Taylor G (2011). Detailed characterization of neuro-immune responses following neuropathic injury in mice. Brain Res..

[29] Kurimoto T (2013). Neutrophils express oncomodulin and promote optic nerve regeneration. J. Neurosci..

[30] Engelhardt JI, Tajti J, Appel SH (1993). Lymphocytic infiltrates in the spinal cord in amyotrophic lateral sclerosis. Arch. Neurol..

[31] Hooten KG, Beers DR, Zhao W, Appel SH (2015). Protective and Toxic Neuroinflammation in Amyotrophic Lateral Sclerosis. Neurotherapeutics.

[32] Valiathan R (2014). Reference ranges of lymphocyte subsets in healthy adults and adolescents with special mention of T cell maturation subsets in adults of South Florida. Immunobiology.

[33] Lee JS, Kim NY, Na SH, Youn YH, Shin CS (2018). Reference values of neutrophil-lymphocyte ratio, lymphocyte-monocyte ratio, platelet-lymphocyte ratio, and mean platelet volume in healthy adults in South Korea. Med. (Baltim.).

[34] Westeneng HJ (2018). Prognosis for patients with amyotrophic lateral sclerosis: development and validation of a personalised prediction model. Lancet Neurol..

[35] Jang JH (2013). Analysis of the C9orf72 hexanucleotide repeat expansion in Korean patients with familial and sporadic amyotrophic lateral sclerosis. Neurobiol. Aging.

